# Sedentarization and Child Health: A Case Study of the Nutritional Status of Children Under 5 Years Old in the Lower Omo Valley, Ethiopia

**DOI:** 10.1002/ajhb.70154

**Published:** 2025-10-09

**Authors:** Sarai M. Keestra, Bereket Yohannes Kabalo, Ehsan Kharati Koopaei, Lucie Buffavand, Tsegaye Demissie Gemebo, Yalew Ayele, Edward G. J. Stevenson

**Affiliations:** ^1^ Pediatric Endocrinology, Emma Children's Hospital Amsterdam the Netherlands; ^2^ Department of Epidemiology and Data Science, Amsterdam UMC University of Amsterdam Amsterdam the Netherlands; ^3^ Amsterdam Reproduction & Development, Amsterdam UMC University of Amsterdam Amsterdam the Netherlands; ^4^ Centre for International Health University of Bergen Bergen Norway; ^5^ School of Public Health Wolaita Sodo University Wolaita Sodo Ethiopia; ^6^ School of Public and Environmental Health Hawassa University Hawassa Ethiopia; ^7^ Department of Mathematical Sciences Durham University Durham UK; ^8^ Manchester Metropolitan University Manchester UK; ^9^ Institut des Mondes Africains Aubervilliers France; ^10^ College of Health Sciences and Medicine, School of Public Health, Department of Reproductive Health and Human Nutrition Wolaita Sodo University Wolaita Sodo Ethiopia; ^11^ International Institute of Rural Reconstruction Jinka Ethiopia; ^12^ Department of Anthropology Durham University Durham UK

**Keywords:** child growth, displacement, nutritional status, pastoralism, resettlement

## Abstract

**Objectives:**

This study evaluates differences in the nutritional status of children under 5 years old among the Bodi (Mela) of southwest Ethiopia, in the context of a sedentarization program which involved resettlement of pastoralist families in government‐designed villages (villagization sites).

**Methods:**

Data were collected in 2013 from two settings: state‐run villagization sites (Hana), where families were forcibly resettled 6–18 months earlier to farm and receive food aid, and comparison communities in cattle camps (Gura). Families with at least one child under 5 years old were recruited. Household characteristics, disease incidence, infant feeding practices, and anthropometric measurements (weight, height, mid‐upper arm circumference, triceps skinfold, and head circumference) were recorded. Age‐adjusted *z*‐scores were calculated and compared between sites. Ethical approval for the study was obtained from Emory University, Wolaita Sodo University, and the Southern Nations, Nationalities and People's Region Health Bureau.

**Results:**

A total of 106 children from 75 families participated (40 in Hana, 35 in Gura). Nearly one‐third of the children were stunted (31.5%) or underweight (27.4%), while 7.6% were wasted. Anthropometric measurements did not differ significantly between the two sites; however, in a sex‐stratified analysis, boys in the villagization site had higher weight‐for‐height but lower triceps skinfold‐for‐age than those in the comparison site. No significant difference in the proportion wasted was observed. Families in Hana were less likely to report their child having consumed animal milk in the past 24 h and more likely to report a case of diarrhea in the past month.

**Conclusions:**

Approximately 1 year after sedentarization, there was no consistent pattern of change in nutritional status among children in resettled families compared to those in pastoralist families.

## 1 Introduction

Pastoralism is an economic system based on the herding of livestock, often entailing mobile or nomadic livelihood (Khazanov 1994). Government administrations, agricultural scientists, and development economists commonly view pastoralism as an inefficient form of production and posit that sedentarization will allow for more stable and efficient livelihoods and improve nutritional status (Sanford 1983; Fratkin 2014). However, a body of empirical research has accumulated running contrary to this prediction. In nomadic groups studied before and after settlement, sedentarization is associated with decreased nutritional status, especially for children (Roth et al. 2005; Shell‐Duncan and Obiero 2000). Decrease in milk consumption and an increase in respiratory illness and diarrhea incidence may be at the root of poorer growth patterns in settled communities (Roth et al. 2005). To investigate this, we examined the nutritional status of children under 5 years of age in a region of Ethiopia in which a sedentarization policy was being implemented.

The Lower Omo in Southwest Ethiopia is home to a diversity of semi‐nomadic groups whose livelihoods depend on a combination of pastoralism, shifting cultivation, and flood‐retreat agriculture along the riverbanks and nearby grasslands, including the Bodi (Mela) (Buffavand 2016; Fukui 2001). Three concurrent state‐led development plans have disrupted the ecology of the region in which the Bodi live. First, a hydroelectric dam built upstream has ended the annual flooding of the river banks, jeopardizing food security and livelihoods of at least 21 000 families downstream (Hodbod et al. 2019; Stevenson et al. 2021). Second, industrial agricultural enterprises, enabled by irrigation from the dam, were created to produce sugarcane for domestic and international markets, displacing people from their lands (Gebeyehu and Abbink 2022; Kamski 2016). Third, a combination of planned and spontaneous migration of farmers and daily laborers from Ethiopia's highland areas to less densely inhabited lowlands has made Bodi a minority group in their own lands (Stevenson and Buffavand 2018). To offset these interventions' negative impacts, villagization projects were initiated in 2012 (Gebresenbet 2021). Anticipated benefits of villagization included improved food security through the adoption of new farming methods and better access to schooling and medical services (FDRE 2012).

## 2 Methods

We included families in two settings: villagization sites (Hana) where people were forced to settle and farm on government land 6–18 months prior to our study, and a comparison community where people were living in cattle camps (Gura). In the villagization site but not in the cattle camps, food aid was distributed by the government. In both settings, we recruited all families with one or more children under 5 years, interviewing the heads of households, usually women, about family characteristics and carrying out anthropometric measurements on children in August–September 2013. After collecting data on household characteristics and family composition, subsistence strategy, and incidence of diarrhea/cough/fever in the past month, household heads were asked if, in the past day, the child drank milk (human or animal), or any (semi) solid foods not eaten by other household members. We analyzed responses at a household level. Weight (kg), height (cm), mid‐upper arm circumference (cm), triceps skinfold (mm), and head circumference (cm) of the children were measured using a Holtain caliper, Seca scale (model 875), Seca stadiometer (model 217), an infant measuring board (provided by the local health department), and Seca 212 tape. Height/length was not recorded for some children < 6 months due to parental discomfort at having their infants laid on a measuring board (*n* = 6 in Gura; *n* = 8 in Hana). We followed the measurement protocol of Cogill (2003). Further information on participant recruitment, enumerator training, and interview methods is available elsewhere (Stevenson and Buffavand 2018, 116–118).

Handwritten data were curated in Excel; statistical analyses were carried out in R version 4.3.2 (R Core Team 2021). Logistic regression was used to estimate odds ratios with 95% CIs for binary outcomes by site; Poisson regression was used for count outcomes and linear regression for continuous outcomes. Age‐ and sex‐adjusted *z*‐scores were derived from WHO Child Growth Standards using the “zscorer” R package, defining stunting, underweight, and wasting as *z* < −2SD for height, weight, and weight‐for‐height (Myatt and Guevarra 2019). Linear regression was used to assess mean differences in anthropometric *z*‐scores, with cluster‐robust standard errors accounting for intra‐household correlations using R packages “sandwich” and “lmtest” (Hothorn et al. 2022; Zeileis et al. 2024), adjusting for the number of children under five in the household, children's age in months, and child sex. We tested interaction terms between site and children's sex to assess potential sex‐specific differences and conducted sex‐stratified analyses (File S2). Ethical approval was obtained from Emory University (2012), Sodo Wolaita University (2013; principal investigator: Craig Hadley), and the Health Bureau of Ethiopia's Southern Nations, Nationalities and People's Region (2013).

## 3 Results

Seventy‐five families were included in the study, 40 at the villagization sites in Hana, and 35 in the cattle camps in Gura. There were no significant differences in family structure based on the number of wives, children under 5, or dependent children under 15 per family, nor were there significant differences in the proportion of households keeping cattle, sheep, or goats, or in the proportion of households in which a parent had ever attended school (Table 1). A higher proportion of households in Hana received income from wage labor. There were no significant differences between households in Hana and Gura regarding children under five being breastfed, drinking water, or consuming solid foods. However, in Hana children were significantly less likely to have drunk animal milk than in Gura in the past 24 h (89% vs. 66%; *p* = 0.03). Children in both sites commonly ate the same foods as adults. There was no difference in household reporting of fever or cough in the past month, but in Hana more households reported at least one member with diarrhea in the past month (60% vs. 34%; *p* = 0.03).

**TABLE 1:** Baseline characteristics on a household level comparing the cattle camps of Gura to the villagization sites in Hana.

	Gura (cattle camps) (*n* = 35)	Hana (villagization sites) (*n* = 40)	Estimated change and 95% confidence interval^a^
Household structure			
Number of household members (mean ± SD)	6.51 (3.18)	5.63 (3.13)	−0.15 (−0.33, 0.04), *p* = 0.12
Number of wives per household (mean ± SD)	1.31 (0.58)	1.60 (0.90)	0.2 (−0.19, 0.58), *p* = 0.31
Number of children under 5 years old (mean ± SD)	1.71 (0.71)	1.75 (0.74)	0.02 (−0.33, 0.37), *p* = 0.91
Subsistence strategies			
Keeping cattle (%)	97.1% [34/35]	75.0% [30/36]	0.15 (0.02, 1.34), *p* = 0.08
Keeping sheep (%)	5.7% [2/35]	5.0% [2/37]	0.94 (0.12, 7.34), *p* = 0.95
Keeping goats (%)	47.5% [19/35]	48.6% [17/37]	1.12 (0.44, 2.86), *p* = 0.81
Engaging in wage labor (%)	**22.9% [8/34]** ^b^	**45.0% [18/36]** ^b^	**3.25 (1.14, 9.25), *p* = 0.02**
Parents ever attended school (%)	17.1% [6/35]	20.0% [8/36]	1.21 (0.37, 3.98), *p* = 0.75
Infant feeding 24‐h recall			
Breastfeeding (%) [*n*]	71.4% [25/35]	82.9% [34/40]	1.94 (0.64, 5.92), *p* = 0.23
Water (%) [*n*]	88.6% [31/35]	90.2% [37/40]	1.19 (0.27, 5.3), *p* = 0.81
Milk (%) [*n*]	**88.6% [31/35]** ^b^	**65.9% [27/40]** ^b^	**0.25 (0.07, 0.86), *p* = 0.03**
Solids (%) [*n*]	82.9% [29/35]	65.9% [27/40]	0.4 (0.13, 1.21), *p* = 0.10
Infectious disease incidence during the last month			
Diarrhea (%) [*n*]	**34.3% [12/35]** ^b^	**60.0% [24/40]** ^b^	**2.87 (1.1, 7.49), *p* = 0.03**
Fever/malaria (%) [*n*]	57.1% [20/35]	45.0% [18/40]	0.61 (0.24, 1.56), *p* = 0.30
Cough (%) [*n*]	57.1% [20/35]	42.5% [17/38]	0.61 (0.24, 1.56), *p* = 0.29

*Note:* Bold indicates significant differences at *p* < 0.05 level.

^a^ Odds ratios were used to calculate the change in percentage between sites in binary variables (e.g., keeping any cattle), rate ratios for changes in count (e.g., number of children under 5 years per family), and linear regression for changes in continuous variables between sites (e.g., age in months).

^b^ Significant differences at *p* < 0.05 level.

From these 75 families, 106 children aged 0–5 years old were included (Hana *n* = 55; Gura *n* = 51), with 55.7% [59/106] girls and 44.3% [47/106] boys. There were no significant differences in age or sex of the children between the sites (File S1). None of the anthropometric measurements differed significantly between Gura and Hana when sexes were combined, but nearly a third of the overall sample was stunted (31.5%) or underweight (27.4%), whereas only 7.6% of the children were wasted (low weight‐for‐height) (Table 2). Interaction terms indicated sex‐specific differences for weight‐for‐height (*β* = 1.01; 95% CI 0.05–1.97; *p* = 0.043) and triceps skinfold *z*‐scores (*β* = −1.41; 95% CI −2.39 to −0.43; *p* = 0.006); in stratified analyses, boys in the villagization site had higher weight‐for‐height *z*‐scores (adjusted estimate = 0.85; 95% CI 0.11–1.58; *p* = 0.030) but lower triceps skinfold‐for‐age *z*‐scores (−1.05; 95% CI −1.91 to −0.18; *p* = 0.023) than those in the comparison site, although no difference was observed in the proportion of boys who were wasted (*z*‐score < −2 for weight‐for‐height) (Table 2; File S2). No significant differences were observed among girls between the two sites.

**TABLE 2:** Age‐adjusted anthropometric measurements comparing 106 children living in Gura and Hana.

	Gura (*n* = 51)	Hana (*n* = 55)	Overall (*n* = 106)	Unadjusted estimate (both sexes) (95% CI), *p*	Adjusted estimate (both sexes) (95% CI), *p* ^a^	Boys only (*n* = 47) adjusted estimate (95% CI), *p* ^a^	Girls only (*n* = 59) adjusted estimate (95% CI), *p* ^a^
Height for age *z*‐scores (mean ± SD)	−1.13 (1.63)	−1.03 (1.66)	−1.08 (1.63)	0.10 (−0.57, 0.76), *p* = 0.776	0.10 (−0.56, 0.77), *p* = 0.756	−0.15 (−1.36, 1.07), *p* = 0.814	0.3 (−0.45, 1.05), *p* = 0.431
Weight for age *z*‐scores (mean ± SD)	−1.21 (1.36)	−1.15 (1.26)	−1.18 (1.31)	0.06 (−0.51, 0.62), *p* = 0.838	0.02 (−0.55, 0.59), *p* = 0.949	0.15 (−0.65, 0.94), *p* = 0.717	−0.05 (−0.64, 0.54), *p* = 0.873
Weight for height *z*‐scores (mean ± SD)	−0.9 (1.1)	−0.66 (1.27)	−0.78 (1.19)	0.24 (−0.27, 0.76), *p* = 0.354	0.27 (−0.29, 0.84), *p* = 0.342	* **0.85 (0.11, 1.58)**, ***p* = 0.030**	−0.16 (−0.84, 0.51), *p* = 0.639
MUAC for age *z*‐scores (mean ± SD)	0.88 (1.15)	0.67 (1)	0.77 (1.07)	−0.21 (−0.65, 0.24), *p* = 0.362	−0.16 (−0.64, 0.32), *p* = 0.507	−0.51 (−1.24, 0.23), *p* = 0.183	−0.01 (−0.51, 0.49), *p* = 0.958
Triceps for age *z*‐scores (mean ± SD)	0.06 (1.4)	−0.11 (0.98)	−0.03 (1.19)	−0.17 (−0.67, 0.33), *p* = 0.502	−0.21 (−0.75, 0.32), *p* = 0.430	* **−1.05 (−1.91, −0.18)**, ***p* = 0.023**	0.36 (−0.16, 0.87), *p* = 0.178
Head circumference for age *z*‐scores (mean ± SD)	0.40 (1.18)	0.44 (1.27)	0.42 (1.22)	0.04 (−0.5, 0.58), *p* = 0.893	0.06 (−0.51, 0.62), *p* = 0.846	0.06 (−0.58, 0.71), *p* = 0.855	−0.02 (−0.69, 0.64), *p* = 0.943
Stunted (< −2SD height‐for‐age) % [*n*/*n*]	33.3% [15/45]	29.8% [14/47]	31.5% [29/92]	−0.04 (−0.24, 0.17), *p* = 0.728	−0.04 (−0.24, 0.16), *p* = 0.670	−0.11 (−0.43, 0.21), *p* = 0.504	−0.01 (−0.26, 0.23), *p* = 0.910
Underweight (< −2SD weight‐for‐age) % [*n*/*n*]	25.5% [13/51]	29.1% [16/55]	27.4% [29/106]	0.04 (−0.16, 0.23), *p* = 0.711	0.05 (−0.13, 0.23), *p* = 0.592	−0.05 (−0.31, 0.20), *p* = 0.692	0.13 (−0.09, 0.35), *p* = 0.239
Wasted (< −2SD weight‐for‐height) % [*n*/*n*]	4.4% [2/45]	10.6% [5/47]	7.6% [7/92]	0.06 (−0.05, 0.17), *p* = 0.259	0.06 (−0.06, 0.17), *p* = 0.220	−0.04 (−0.21, 0.13), *p* = 0.676	0.13 (−0.03, 0.29), *p* = 0.113

* *Note:* Bold indicates significant differences at *p* < 0.05 level.

^a^ Estimates are from linear regression models and for all variables, except percentage of girls/boys and age in months, include cluster‐robust standard errors to account for household‐level clustering. For the adjusted estimates, child sex (in the full sample, not in the sex‐stratified analysis), child age in months, and the number of children under five in the household were used as covariates. For percentage of girls/boys and difference per site, an unadjusted odds ratio was used.

## 4 Discussion

Governments in Eastern Africa have long pursued policies of sedentarization, encouraging—and in some cases forcing—pastoralists to abandon their traditional ways of life and to settle permanently either as farmers or townspeople (Fratkin 2014). The implications of these policies for nutrition and health are disputed. In this study, we found no improvement in the nutritional status of children under five approximately 1 year after resettlement. Corresponding with previous studies, we found that when pastoralists are forced to settle, this may increase the incidence of diarrhea and decrease milk consumption among children under five, both of which can affect their growth and nutritional status in the long term (Bogin et al. 2007; Kabalo and Lindtjørn 2022; Roth et al. 2005; Wells et al. 2017). Previous research in Ethiopia has furthermore shown that boys are more vulnerable to undernutrition in early life than girls (Medhin et al. 2010). In accordance with this, we found no significant anthropometric differences among girls, but some differences among boys; specifically, we found higher weight‐for‐height but lower triceps skinfolds (an indicator of subcutaneous fat) among boys in villagization sites approximately 1 year post‐resettlement compared to those living in semi‐nomadic cattle camps. This may be accounted for by differences in diet between the two sites: in the villagization sites, households received food aid consisting mainly of maize; there was lower cattle ownership and greater distance to land where cattle were kept. In the cattle camps of Gura, by contrast, more children drank animal milk, an important source of fat in early life for pastoralist children (Roth et al. 2005; Ferro‐Luzzi et al. 2001).

Despite improving food security being a central aim of villagization, we previously reported that moderate and severe food insecurity remained high in Hana (85%) and Gura (97%), with slightly lower food insecurity in villagization sites likely reflecting state‐provided food aid rather than improved resilience among resettled communities (Stevenson and Buffavand 2018). Ethnographic data collected alongside these surveys revealed further challenges to livelihoods in villagization sites, where difficulties in maintaining cattle husbandry led to reduced milk access, forcing families to depend on market purchases to meet their children's nutritional needs (Stevenson et al. 2025). This case study highlights how sedentarization can disrupt pastoralist dietary practices such as children's consumption of animal milk, which is an important source of fat, protein, and micronutrients, underscoring the need for interventions that account for pastoralists' ecological and cultural contexts (Roth et al. 2005; Ferro‐Luzzi et al. 2001). Indeed, holding livestock can buffer children from seasonal variation in undernutrition and wasting (low weight‐for‐height) in particular (Ferro‐Luzzi et al. 2001).

This study has several limitations. Collection of data at the household level as opposed to the level of individual children limited our ability to adjust feeding and illness variables for individual child age, as some families included multiple children under 5 years old. The small sample size limited our ability to detect the significance of differences between groups where effect sizes were small. While we used age‐adjusted WHO standards for comparability, linear growth rates might differ between East African pastoralist children and those children measured for the Multicentre Growth Reference Study, on which the WHO growth standards are based (de Onis et al. 2004; Little and Johnson 1987; Marume et al. 2022). Furthermore, stunting and being underweight do not always reflect undernutrition per se but could reflect structural disadvantage (Hermanussen et al. 2022; Scheffler et al. 2020), which in the Lower Omo is shaped by systemic marginalization of pastoralist communities through displacement and disruption of livelihoods (Stevenson and Buffavand 2018). As child wasting varies with seasonal household food insecurity (Kabalo and Lindtjørn 2022), differences in nutritional status may emerge seasonally and may not be adequately captured in our cross‐sectional study. Additionally, data collection occurred approximately 1 year after resettlement, but the duration of residence in the villagization sites varied by household. Given the short interval between resettlement and measurement, it is possible that not enough time had passed to observe catch‐up growth. Furthermore, concurrent ethnographic research indicated that families moved in and out of these sites in response to food aid availability and livelihood opportunities elsewhere (Stevenson and Buffavand 2018). Within 5 years, the villagization project was abandoned due to intergroup conflict and the poor quality of farmland and irrigation infrastructure in the resettlement sites (Stevenson et al. 2021).

Overall, this case study supports a body of evidence that forced sedentarization of nomadic people does not necessarily improve children's nutritional status, at least not in the short term. In order to yield benefits for nutritional status and growth of under‐5‐year‐olds, sedentarization programs must be accompanied by sustainable long‐term investment in the health, security, and wellbeing of children in this important developmental period.

## Author Contributions

**Sarai M. Keestra:** data curation (lead), formal analysis (lead), writing – original draft preparation (lead), writing – review and editing (equal). **Bereket Yohannes Kabalo:** conceptualization (equal), data curation (equal), investigation (equal), project administration (supporting), writing – review and editing (equal). **Ehsan Kharati Koopaei:** formal analysis (supporting). **Lucie Buffavand:** conceptualization (equal), funding acquisition (supporting), writing – review and editing (equal). **Tsegaye Demissie Gemebo:** conceptualization (equal), data curation (equal), methodology (supporting), writing – review and editing (equal), resources (supporting). **Yalew Ayele:** investigation (equal), project administration (supporting), writing – review and editing (supporting). **Edward G. J. Stevenson:** conceptualization (lead), funding acquisition (lead), methodology (lead), project administration (lead), supervision (lead), writing – review and editing (equal).

## Conflicts of Interest

Sarai M. Keestra is a member of People's Health Movement and the WHO Watch. The other authors report no conflicts of interest.

## Supporting information


**File S1:** ajhb70154‐sup‐0001‐FileS1.docx.

**File S2:** ajhb70154‐sup‐0001‐FileS2.docx.

## Data Availability

The data that support the findings of this study are available from the corresponding author upon reasonable request.
