# Supplementary material for: Sedentarization and Child Health: A Case Study of the Nutritional Status of Children Under 5 Years Old in the Lower Omo Valley, Ethiopia
Source: Am J Hum Biol. 2025 Oct 9;37(10):e70154. doi: 10.1002/ajhb.70154 (PMC12509176; doi:10.1002/ajhb.70154)
Supplement: Supplementary file 1 — File S1: ajhb70154‐sup‐0001‐FileS1.docx. [file AJHB-37-e70154-s002.docx]

Supplementary File 1: Age distribution and sex of children under five years old in Gura and Hana

| **Age Group** | **Gura** | **Hana** | **Overall** | **Hana – Gura Difference (%) (p-value)** |
| --- | --- | --- | --- | --- |
| 0 to 1years | 15.7% [8/51] | 32.7% [18/55] | 24.5% [26/106] | 17% (p=0.07) |
| 1 to 2 years | 23.5% [12/51] | 12.7% [7/55] | 17.9% [19/106] | -10.8% (p=0.232) |
| 2 to 3 years | 19.6% [10/51] | 21.8% [12/55] | 20.8% [22/106] | 2.2% (p=0.968) |
| 3 to 4 years | 11.8% [6/51] | 20% [11/55] | 16% [17/106] | 8.2% (p=0.374) |
| 4 to 5 years | 29.4% [15/51] | 12.7% [7/55] | 20.8% [22/106] | -16.7% (p=0.061) |


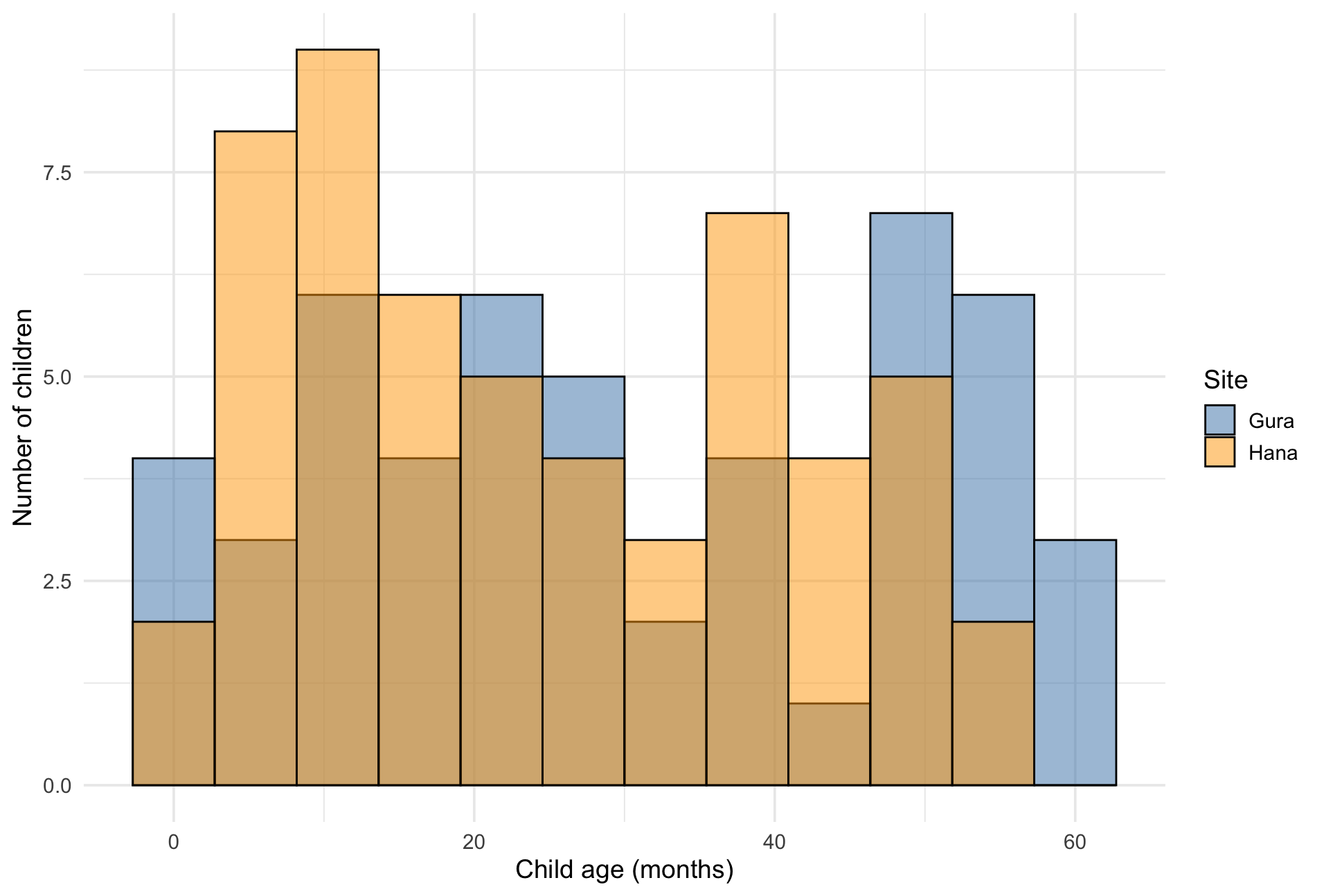


|  | Gura  (Cattle Camps) (n=51) | Hana  (Villagization Sites) (n=55) | Overall (n=106) | Unadjusted estimate (both sexes) (CI95%),  p-value |
| --- | --- | --- | --- | --- |
| Percentage of girls (%) [n] | 54.9% [28/51] | 56.4% [31/55] | 55.7% [59/106] | 1.06 (0.49, 2.28),  p = 0.880 |
| Percentage of boys (%) [n] | 45.1% [23/51] | 43.6% [24/55] | 44.3% [47/106] | 0.94 (0.44, 2.03),  p = 0.88 |
| Age in months median [IQR] | 27 [14-48] | 24 [10-37] | 24 [12-44] | -5.12 (-11.72, 1.47), p = 0.131 |
