# Supplementary material for: Sedentarization and Child Health: A Case Study of the Nutritional Status of Children Under 5 Years Old in the Lower Omo Valley, Ethiopia
Source: Am J Hum Biol. 2025 Oct 9;37(10):e70154. doi: 10.1002/ajhb.70154 (PMC12509176; doi:10.1002/ajhb.70154)
Supplement: Supplementary file 2 — File S2: ajhb70154‐sup‐0001‐FileS2.docx. [file AJHB-37-e70154-s001.docx]

Supplementary File 2: Sex-stratified analysis

Interaction term

| Variable | Interaction term Site x Sex (95%CI), p.value † |
| --- | --- |
| Height for age z-scores (mean±SD) | -0.46 (-1.9, 0.97), p = 0.53 |
| Weight for age z-scores (mean±SD) | 0.22 (-0.77, 1.22), p = 0.663 |
| Weight for height z-scores (mean±SD) | **1.01 (0.05, 1.97), p = 0.043** |
| MUAC for age z-scores (mean±SD) | -0.49 (-1.3, 0.32), p = 0.234 |
| Triceps for age z-scores (mean±SD) | **-1.41 (-2.39, -0.43), p = 0.006** |
| Head circumference for age z-scores (mean±SD) | 0.05 (-0.81, 0.91), p = 0.905 |
| Stunted (<-2SD height-for-age) % [n/n] | -0.02 (-0.4, 0.35), p = 0.909 |
| Underweight (<-2SD weight-for-age) % [n/n] | -0.17 (-0.49, 0.15), p = 0.303 |
| Wasted (<-2SD weight-for-height) % [n/n] | -0.16 (-0.37, 0.05), p = 0.136 |

†interaction term results are adjusted for household size (under-fives) and account for clustering at the family level.

Girls

|  | **Gura  (cattle camp) (n=28)** | **Hana (resettlement site) (n=31)** | **Overall (n=59)** | **Unadjusted estimate (CI95%),  p-value** | **Adjusted estimate (CI95%),  p-value**  **†** |
| --- | --- | --- | --- | --- | --- |
| Height for age z-scores (mean±SD) | -1.18 (1.43) | -0.75 (1.44) | -0.96 (1.44) | 0.42 (-0.34, 1.19), p = 0.283 | 0.3 (-0.45, 1.05), p = 0.431 |
| Weight for age z-scores (mean±SD) | -1.13 (1.07) | -1.08 (1.29) | -1.1 (1.18) | 0.06 (-0.55, 0.66), p = 0.858 | -0.05 (-0.64, 0.54), p = 0.873 |
| Weight for height z-scores (mean±SD) | -0.6 (0.9) | -0.72 (1.43) | -0.66 (1.18) | -0.12 (-0.76, 0.52), p = 0.712 | -0.16 (-0.84, 0.51), p = 0.639 |
| MUAC for age z-scores (mean±SD) | 0.73 (1) | 0.71 (0.87) | 0.72 (0.93) | -0.02 (-0.51, 0.46), p = 0.923 | -0.01 (-0.51, 0.49), p = 0.958 |
| Triceps for age z-scores (mean±SD) | -0.25 (1.19) | 0.17 (0.77) | -0.03 (1.01) | 0.42 (-0.1, 0.94), p = 0.115 | 0.36 (-0.16, 0.87), p = 0.178 |
| Head circumference for age z-scores (mean±SD) | 0.33 (1.12) | 0.31 (1.43) | 0.32 (1.28) | -0.02 (-0.69, 0.64), p = 0.945 | -0.02 (-0.69, 0.64), p = 0.943 |
| Stunted (<-2SD height-for-age) % [n/n] | 29.6% [8/27] | 22.2% [6/27] | 25.9% [14/54] | -0.07 (-0.31, 0.16), p = 0.542 | -0.01 (-0.26, 0.23), p = 0.910 |
| Underweight (<-2SD weight-for-age) % [n/n] | 21.4% [6/28] | 29% [9/31] | 25.4% [15/59] | 0.08 (-0.15, 0.3), p = 0.508 | 0.13 (-0.09, 0.35), p = 0.239 |
| Wasted (<-2SD weight-for-height) % [n/n] | 3.7% [1/27] | 14.8% [4/27] | 9.3% [5/54] | 0.11 (-0.04, 0.27), p = 0.164 | 0.13 (-0.03, 0.29), p = 0.113 |

**†:** estimates are from linear regression models and include cluster-robust standard errors to account for household-level clustering. For the adjusted estimates child age in months, and the number of children under five in the household were used as covariates.

Boys

|  | **Gura  (cattle camp) (n=23)** | **Hana (resettlement site) (n=24)** | **Overall (n=47)** | **Unadjusted estimate (CI95%),  p-value** | **Adjusted estimate (CI95%),  p-value**  **†** |
| --- | --- | --- | --- | --- | --- |
| Height for age z-scores (mean±SD) | -1.05 (1.92) | -1.41 (1.89) | -1.24 (1.89) | -0.35 (-1.56, 0.85), p = 0.568 | -0.15 (-1.36, 1.07), p = 0.814 |
| Weight for age z-scores (mean±SD) | -1.30 (1.67) | -1.25 (1.25) | -1.27 (1.46) | 0.06 (-0.79, 0.9), p = 0.896 | 0.15 (-0.65, 0.94), p = 0.717 |
| **Weight for height z-scores (mean±SD)** | **-1.36 (1.24)** | **-0.58 (1.04)** | **-0.95 (1.19)** | **0.78 (0.05, 1.5), p = 0.044** | **0.85 (0.11, 1.58), p = 0.03** |
| MUAC for age z-scores (mean±SD) | 1.1 (1.33) | 0.63 (1.18) | 0.84 (1.26) | -0.47 (-1.23, 0.29), p = 0.235 | -0.51 (-1.24, 0.23), p = 0.183 |
| **Triceps for age z-scores (mean±SD)** | **0.52 (1.58)** | **-0.49 (1.12)** | **-0.02 (1.43)** | **-1.01 (-1.86, -0.17), p = 0.024** | **-1.05 (-1.91, -0.18), p = 0.023** |
| Head circumference for age z-scores (mean±SD) | 0.49 (1.27) | 0.61 (1.03) | 0.55 (1.14) | 0.12 (-0.56, 0.8), p = 0.732 | 0.06 (-0.58, 0.71), p = 0.855 |
| Stunted (<-2SD height-for-age) % [n/n] | 38.9% [7/18] | 40% [8/20] | 39.5% [15/38] | 0.01 (-0.31, 0.33), p = 0.946 | -0.11 (-0.43, 0.21), p = 0.504 |
| Underweight (<-2SD weight-for-age) % [n/n] | 30.4% [7/23] | 29.2% [7/24] | 29.8% [14/47] | -0.01 (-0.28, 0.25), p = 0.926 | -0.05 (-0.31, 0.2), p = 0.692 |
| Wasted (<-2SD weight-for-height) % [n/n] | 5.6% [1/18] | 5% [1/20] | 5.3% [2/38] | -0.01 (-0.15, 0.14), p = 0.941 | -0.04 (-0.21, 0.13), p = 0.676 |

**†:** estimates are from linear regression models and include cluster-robust standard errors to account for household-level clustering. For the adjusted estimates child age in months, and the number of children under five in the household were used as covariates.
